# Anastomotic Openings between Hollow Viscera

**Published:** 1901-06-01

**Authors:** 


### ANASTOMOTIC OPENINGS BETWEEN HOLLOW VISCERA.

Addressing the American Surgical Association on the subject of the surgical treatment of chronic ulcer of the stomach, Mr. Mayo Robson referred to a method which he has systematically pursued since 1891, not only in stomach operations but in nearly all operations involving the making of an anastomotic opening between hollow viscera—the method of suture over a decalcified bone bobbin. This plan he has adopted in a very large number of cases and in a great variety of operations, and as a result of this extensive experience he is more than ever convinced that it is a reliable procedure which can be thoroughly recommended. It is really very simple and only involves two continuous sutures, one of chromicised catgut to unite the mucous margins of the two openings, and one of celluloid thread to unite the serous surfaces about a quarter of an inch away from the new opening. This Pagenstecher's thread or spun celluloid has replaced silk in his practice, being stronger, easily sterilised by boiling, and less absorbent. The decalcified bone bobbin (made by Messrs. Down Brothers in various sizes) is nothing more than a hollow cylinder of decalcified bone, with raised ends, which is placed in the new anastomotic opening around which the sutures are applied. The advantages claimed for the method are that it secures the opening being of the exact size intended, and that there is no possibility of its being made too small by the drawing up of the sutures before the knots are tightened; that it secures an immediately patent channel; that the bobbin protects the new line of union from pressure and irritation for from 24 to 48 hours; that it makes the application of the sutures easier than would otherwise be the case and thus expedites the operation; that as the bobbin rapidly dissolves in the alimentary juices no foreign body is left to cause subsequent trouble; and lastly, that ample experience has shown the method to be rapid, efficient, and safe. In employing this method it is convenient to begin with the serous suture, which is applied around the posterior half of the circle; the needle, still threaded, is then laid aside till the final stage; the openings into the viscera are then made and any redundant mucous membrane is cut away; when this is done the mucous suture is applied to the posterior half of the circle; the bone bobbin is now inserted and closed in by the mucous suture being carried round to where the mucous stitch was begun and where the loose end will be found, to which the end of the catgut is now to be united; the serous suture is now picked up and continued from where it ceased, around the anterior half of the circle, until it meets the loose end of the celluloid thread when the two ends are firmly tied. There can, we think, be no doubt as to the advantages of this method. The decalcified bone bobbin is a distinct advantage in affording protection and rest to the "join" during early hours of union; it soon dissolves when its work is done, and no one can use it without appreciating the increased facility which it affords for exactness of work in applying the sutures, and such exactness, with well-apportioned distribution of tension, goes for much in anastomotic operations.

